# A New Procedure for Ultrasound-Guided Hydrorelease for the Scarring After Arthroscopic Knee Surgery

**DOI:** 10.7759/cureus.12405

**Published:** 2020-12-31

**Authors:** Takahiro Machida, Akihisa Watanabe, Shinichi Miyazawa

**Affiliations:** 1 Rehabilitation, Machida Orthopaedics, Kōchi, JPN; 2 Intelligent Orthopaedic System Development, Okayama University Graduate School of Medicine, Dentistry and Pharmaceutical Science, Okayama, JPN

**Keywords:** after arthroscopy, anterior knee pain, infrapatellar fat pad, postoperative scarring, ultrasound, hydrorelease

## Abstract

Postoperative scarring is one of the complications after arthroscopic knee surgery, which is usually treated with manual therapy or arthroscopic debridement. The incidence of symptomatic scarring requiring surgery within six months postoperatively has been reported to be approximately 0.06-6.00%. We treated a patient after arthroscopic surgery with a new “ultrasound-guided hydrorelease” procedure and we describe it. A 50-year-old woman with a history of arthroscopic anterior cruciate ligament reconstruction of the right knee presented to our clinic 10 months ago with a complaint of right anterior knee pain. Ultrasound imaging showed an infrapatellar fat pad (IPFP) scarring and sliding defects. The pre-treatment Kujala scale was 62 points and the visual analogue scale was 72. The inferolateral side of the patella was palpated to identify the scarring after arthroscopy at the IPFP area. An ultrasound probe was applied vertically to identify the site of adhesion of interest. Ultrasound-guided hydrorelease was performed using 7.0mL saline injected by needle (22G, 60mm) directed at the site with hypoechoic changes in the IPFP. After the procedure, the normalization of the IPFP sliding was confirmed by an ultrasound image. Four weeks after this procedure, the patient improved, with a Kujala scale of 82 points and a visual analogue scale of 28. The most important finding from this patient's course is that her chief complaint of anterior knee pain improved by ultrasound-guided hydrorelease into the IPFP scarring after arthroscopic knee surgery. The procedure should be considered as a treatment for scarring after arthroscopic knee surgery.

## Introduction

Postoperative scarring is one of the complications after arthroscopic knee surgery. This results in increased fibrosis in the infrapatellar fat pad (IPFP) [1], which contributes to a lack of terminal extension, anterior knee pain (AKP), and decrease patellar mobility [2]. Fascial manual therapeutic techniques [3] and anterior interval release surgery [4,5] have been proposed as treatments for this problem, and it has been reported that the rate of symptomatic scarring requiring surgery within six months postoperatively is approximately 0.06-6.00% [6]. This incidence increases as the number of surgeries or the complexity of the surgical procedures increases. Developing a less invasive treatment than surgery for this problem is relevant to both patients and clinicians.

The ultrasound-guided intervention has been applied clinically, and the effectiveness of ultrasound-guided nerve blocks [7] or injections releasing physiological saline to the surrounding soft tissues (often described as “hydrorelease”) has been reported [8]. Hydrorelease is defined as using a few saline injections directed at the site with hyperechoic changes in the soft tissues [8]. We treated a patient after an arthroscopic knee surgery using ultrasound-guided hydrorelease of the scarring in the IPFP. We report here the patient course and describe the technique for the ultrasound-guided hydrorelease.

Ethical approval is not applicable, but written consent was obtained from the patient for publication of the details and images from the case. Written permission was obtained from the subject for publication of this case report.

## Case presentation

**Figure 1:**
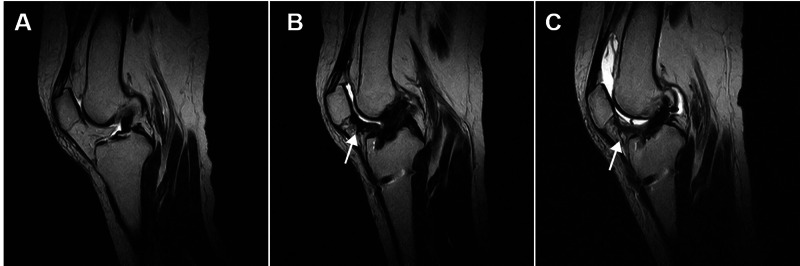
Magnetic resonance imaging (A) Pre-arthroscopic anterior cruciate ligament reconstruction -- indicates anterior cruciate ligament tear. (B) Post-surgery -- the anterior cruciate ligament continuity is confirmed. The scar of the infrapatellar fat pad (arrow). (C) Ten months after surgery -- the anterior cruciate ligament is not re-tearing. The scar of the infrapatellar fat pad (arrow).

A 50-year-old woman presented to our primary care clinic in Japan with a chief complaint of AKP. She had undergone arthroscopic anterior cruciate ligament (ACL) reconstruction of her right knee 10 months previously (Figure 1) and was undergoing postoperative rehabilitation, including manual therapy (percutaneous traction on scars) described by Alvira-Lechuz et al., range of motion exercises, and strength exercises [3]. However, the AKP persisted long after the surgery and the pain made it difficult for her to descend the stairs.

**Figure 2:**
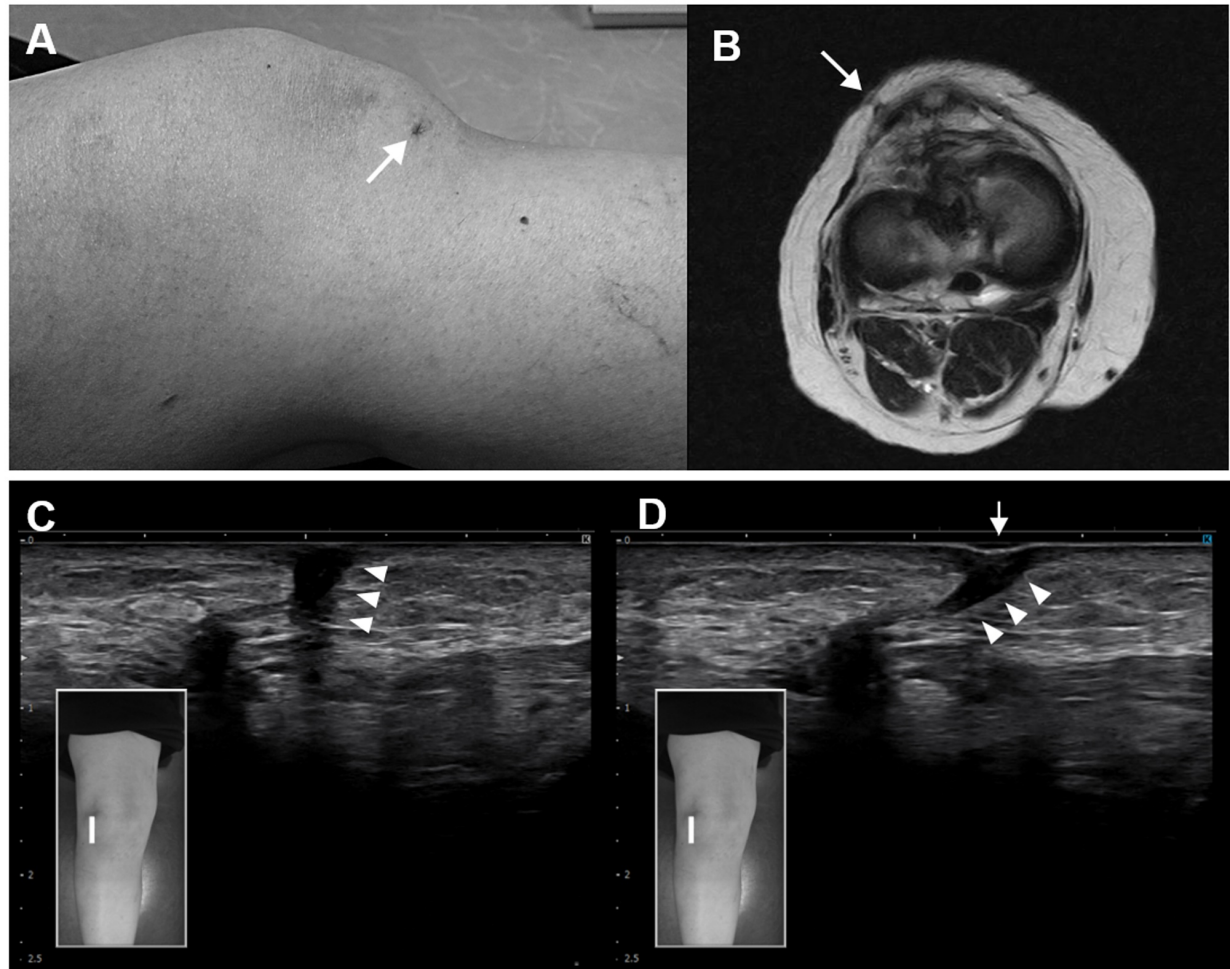
Postoperative scarring (A) Exterior view of the right knee -- scar on the patient’s right knee (arrow). (B) Magnetic resonance imaging -- scar of the skin incision from the previous arthroscopic surgery (arrow). (C) Ultrasound imaging -- hypoechoic in the scar area (arrowhead). (D) The scar area is not sliding (arrowhead) with manual sliding of the skin. The surface of the skin is deeply retracted and concaved (arrow).

Magnetic resonance imaging confirmed that there was no re-tearing of the reconstructed ACL but showed a scar from the skin incision from the previous arthroscopic surgery (Figure 2). Lachman’s test was negative, and there was no instability of the knee. The range of motion of the knee showed an active extension of -5˚ and an active flexion of 140˚ (contralateral, 0˚ to 150˚), as well as a loss of patellar mobility in supra-inferior directions. Palpation on the scar was painful, suggesting limited mobility of formed fibrotic tissue. The Kujala scale was 62 points and the visual analogue scale (VAS) during descending the stairs was 72mm [9]. The Knee Injury and Osteoarthritis Outcome Score (KOOS) showed KOOS pain of 69.4, KOOS symptoms of 75.0, and KOOS ADL of 94.1 [10]. Ultrasound imaging showed the IPFP scarring and sliding defects (Figure 2). She was diagnosed with patellofemoral pain syndrome and treated as follows.

**Figure 3:**
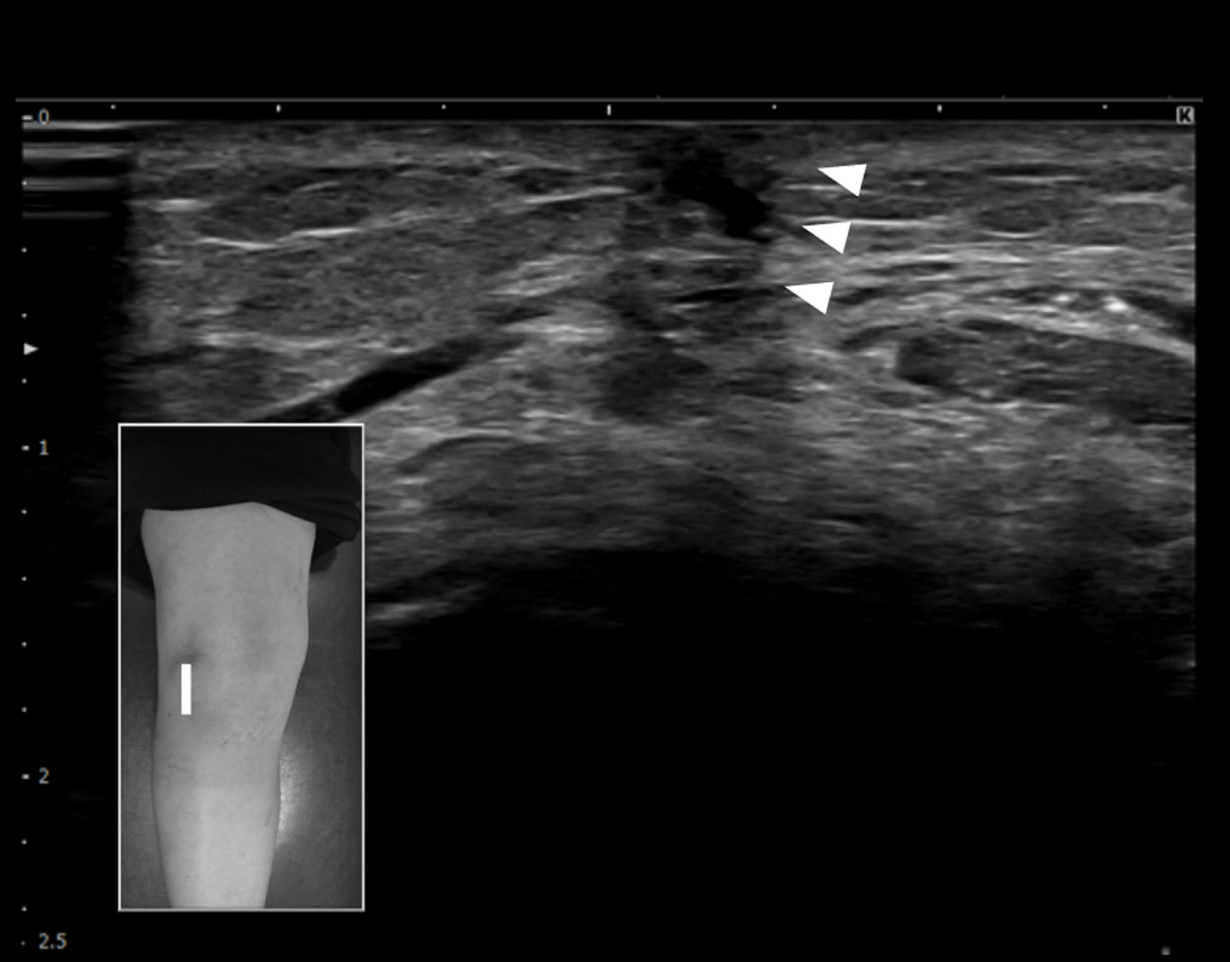
After ultrasound-guided hydrorelease The hypoechoic in the scar area (arrowhead) is attenuated and sliding is improved when the skin is manually slid. The surface of the skin is no longer pulled deep.

The patient was lying on the treatment bed in a supine position, with the knee joint at 30˚ of flexion. The inferolateral side of the patella was palpated to identify the scarring after arthroscopy at the IPFP area. Ultrasound was performed using a 4-18MHz B-mode linear array scanner (SONIMAGE HS2, Konica Minolta, Tokyo, Japan). The probe was applied vertically to identify the adhesion area of interest. Ultrasound-guided hydrorelease was performed using 7.0mL saline injected with a needle (22G, 60mm) directed at the site within 10 mm depth of hypoechoic changes in the IPFP (Figure 3). The images pre-and post-injection were compared to identify the sliding of the IPFP. The ultrasound-guided hydrorelease was performed only once, and the treatment time was about 10 minutes, including ultrasound image examination. All procedures were performed by one orthopedic surgeon with more than 10 years of experience.

After ultrasound-guided hydrorelease, the patient was given rehabilitation including manual therapy (percutaneous traction on scars), mobilization of the patella, and strength exercises for the quadriceps femoris muscle for four weeks.

After four weeks, the Kujala scale was 82 points, and the VAS score during descending the stairs was 28mm. KOOS improved only slightly, with KOOS pain of 75.0, KOOS symptoms of 75.0, and KOOS ADL of 98.5. However, the items related to stair descent improved (P6, “going up or downstairs” and A1, “descending stairs”). The range of motion of the knee showed an active extension of 0˚, and the mobility of the patellar was no longer limited. The normalization of the IPFP sliding was confirmed by ultrasound image.

The patient had moderate needle prick pain (seven of 10 on an 11-point numerical rating pain scale) while undergoing ultrasound-guided hydrorelease. She felt a heaviness in her right knee for two days after the procedure, which improved spontaneously without specific treatment. There was no recurrence of heaviness thereafter.

## Discussion

The most important finding from this patient's course is that her chief complaint of AKP and painful stair descent improved by ultrasound-guided hydrorelease into the IPFP scarring after arthroscopic knee surgery.

Treatment of this patient’s IPFP reduced her pain during stair descent. The patient had pain when descending the stairs, but four weeks after ultrasound-guided hydrorelease, she was able to descend the stairs with little pain. As for the patient’s KOOS, most of the items did not change, but those related to stair descent improved. The minimal clinically important difference on the Kujala scale was 10, and the patient's Kujala scale improved by 20 points, which is a clinically meaningful improvement [11]. Previous studies have reported that irritation of the IPFP is a potential source of AKP [12], that the histopathological changes in IPFP are associated with patellofemoral disorders [13-15], and that pain during stair descent is characteristic of patellofemoral disorders [15-19].

Ultrasound-guided hydrorelease is often performed for myofascial pain syndrome, focusing on the interfascia between the muscles [20]. To the best of our knowledge, no study has applied this technique to postoperative scar tissue and/or IPFP. Although the analgesic mechanism of hydrorelease is not well understood, the following mechanisms have been postulated: (1) washout of the various algesic substances in the interfascial space; (2) decreasing of the viscosity of interfascial fluid; and (3) separation of the myofascial layers which reduces muscular friction resulting in a smooth movement [8]. In this study, we hypothesized that the mechanisms of tissue separation, which reduces tissue friction and provides smooth movement, may have improved the sliding of the IPFP. After ultrasound-guided hydrorelease, this patient had improved patellar mobility and active extension range of motion, which could also corroborate the improvement in sliding of the IPFP [5]. Hence, these mechanisms may have improved AKP and stair descent, which were derived from the mobility of the patellofemoral joint. Ultrasound confirmation that the IPFP is sliding better will be the key to the success of this procedure. Furthermore, this clinical experience has the potential to contribute to future basic biomechanical research, including on IPFP or patellofemoral joint dynamics.

Ultrasound-guided hydrorelease is more effective than manual therapy and has the potential to be a less invasive procedure than surgery. Previous studies have reported the effectiveness of manual therapy [3] and surgery for scarring after arthroscopic knee surgery [4,5]. Although the patient had been undergoing manual therapy, the knee pain had not improved [3]. This procedure should be considered as an optional treatment for patients that have resisted several months of manual therapy for scarring after arthroscopic knee surgery. Furthermore, we believe that clinicians may perform less invasive ultrasound-guided hydrorelease procedures on patients before considering surgery.

The ultrasound-guided hydrorelease resulted in a feeling of heaviness for two days. In a previous study using hydrorelease, although no serious adverse reactions were observed, some participants experienced pain described as a heavy feeling around the injection area with physiological saline injection [8]. An explanation of the adverse events regarding ultrasound-guided hydrorelease would be useful when obtaining informed consent from patients. Further investigation of the incidence of adverse events in ultrasound-guided hydrorelease is needed.

## Conclusions

In conclusion, the patient who was unable to descend the stairs due to AKP improved by ultrasound-guided hydrorelease into the IPFP scarring after arthroscopic knee surgery. This procedure should be considered as a treatment for these scarring lesions. The effectiveness of ultrasound-guided hydrorelease in other patients and other types of scar tissue needs to be investigated, which would contribute to a deeper understanding of the strengths and weaknesses of this procedure.
